# Use of catheter with 2-methacryloyloxyethyl phosphorylcholine polymer coating is associated with long-term availability of central venous port

**DOI:** 10.1038/s41598-021-84885-4

**Published:** 2021-03-08

**Authors:** Yuuki Iida, Kumiko Hongo, Takanobu Onoda, Yusuke Kita, Yukio Ishihara, Naoki Takabayashi, Ryo Kobayashi, Takeyuki Hiramatsu

**Affiliations:** Department of Surgery, Yaizu City Hospital, 1000 Dobara, Yaizu City, Shizuoka 425-8505 Japan

**Keywords:** Biotechnology, Diseases, Medical research

## Abstract

Central venous port (CVP) is a widely used totally implantable venous access device. Recognition of risks associated with CVP-related complications is clinically important for safe, reliable, and long-term intravenous access. We therefore investigated factors associated with CVP infection and evulsion, including the device type. A total of 308 consecutive patients with initial CVP implantation between January 2011 and December 2017 were retrospectively reviewed, and the association of clinical features with CVP-related complications were analyzed. Intraoperative and postoperative complications occurred in 11 (3.6%) and 39 (12.7%) patients, respectively. The overall rate of CVP availability at six months was 91.4%. Malignancy and 2-Methacryloyloxyethyl phosphorylcholine (MPC) polymer-coated catheter use were negatively associated with the incidence of CVP infections. Accordingly, malignancy and MPC polymer-coated catheter use were independent predictors for lower CVP evulsion rate (odds ratio, 0.23 and 0.18, respectively). Furthermore, both factors were significantly associated with longer CVP availability (hazard ratio, 0.24 and 0.27, respectively). This retrospective study identified factors associated with CVP-related complications and long-term CVP availability. Notably, MPC polymer-coated catheter use was significantly associated with a lower rate of CVP infection and longer CVP availability, suggesting the preventive effect of MPC coating on CVP infection.

## Introduction

Treatment with intravenous administration of various chemotherapeutic agents has been increasing in parallel with the increasing number of patients with malignancies. These chemotherapeutic regimens require safe and effective methods for intravenous administration. Home parenteral nutrition, a method of central venous nutrition in patients with terminal cancer or short bowel syndrome, requires reliable access to central veins as well. Although frequently used for intravenous administration, peripheral intravenous catheters are not very safe and reliable. Some agents induce angialgia, and extravasation of the administered agent may result in skin ulcer or necrosis due to toxicity to local tissue. Thus, central venous ports (CVPs), totally implantable venous access devices that have been increasingly used since 1982, are utilized widely to perform chemotherapy, home parenteral nutrition, and blood transfusion^1^. CVP is considered a safe and reliable approach for long-term access to central veins.

Mortality related to CVP implantation is relatively rare (< 2%)^2,3^; however, the procedure is associated with several potential issues including intraoperative complications, such as arterial bleeding and pneumothorax, and postoperative complications, such as infection, occlusion, thrombosis, extravasation (port leakage), and catheter fracture (pinch-off)^1^. Some complications result in unnecessary surgery for evulsion or replacement of the CVP, lead to lower quality of life, or extend the administration of chemotherapy or parenteral nutrition. Although various surgical approaches and devices have been developed to overcome these complications, the complications accounts for up to 33%^1^. Several reports have suggested that the use of CVPs for patients receiving parenteral nutrition or for those with benign diseases are risk factors for infection or evulsion^4–6^; however, contribution of other factors, such as the type of surgical approach and the type of device, to the observed increased risk of CVP use has not been extensively elucidated. Therefore, we aimed to determine risk factors associated with CVP infection or evulsion, particularly the contribution of CVP device type.

## Results

The clinical features of the study patients are presented in Table 1. The patient age ranged from 25 to 97 years (mean age, 69.0 ± 11.5 years). Of the 308 patients included in the study, the primary disease was malignancy in 272 patients (88.3%) and benign in 36 patients (11.7%). Patients with malignancies received CVP implantation for either chemotherapy (n = 211) or nutrition (n = 61), while all the patients with benign disease received the implantation for nutrition. The status of primary disease is strongly associated with purpose of CVP implantation and is clinically definite preoperatively compared to purpose of the implantation, therefore we included the status of primary disease for the following multivariate analyses. The approach involved the internal jugular vein and the subclavian vein in 217 (70.5%) and 86 (27.9%) patients, respectively, and Orphis CV Kit Neo and Bard X-Port isp were used for CVP placement in 176 (57.1%) and 101 (32.8%) patients, respectively.

**Table 1:** Patient characteristics.

Patient characteristics	Number	%
**Gender**		
Female	159	51.6
Male	149	48.4
Mean age (years ± SD)	69.0 ± 11.5	
**Primary disease**		
Benign	36	11.7
Malignant	272	88.3
**Purpose**		
Nutrition	97	31.5
Chemotherapy	211	68.5
**Puncture site**		
Internal jugular vein	217	70.5
Subclavian vein	86	27.9
Others	5	1.6
**Port device**		
Bard X-Port isp	101	32.8
Orphis CV Kit Neo	176	57.1
Others	31	10.1

*SD* standard deviation.

The intraoperative and postoperative complications are summarized in Table 2. The intraoperative complications, which developed in 11 (3.6%) patients, included arterial bleeding and pneumothorax in 7 (2.2%) and 4 (1.3%) patients, respectively. The patients with arterial bleeding required transient hemostasis, and those with pneumothorax needed chest drainage. However, there were no CVP-implantation-related mortalities throughout the study period. The intraoperative complications were not associated with the background characteristics of the patients, puncture site, or the CVP device type (data not shown). The postoperative complications included infection (n = 27, 8.8%), extravasation (n = 5, 1.6%), and others (n = 7, 2.2%) (Table 2). The incidence of CVP infections, the most frequent complication resulting in CVP evulsion, was associated with the primary disease type and the CVP device type. Specifically, the incidence of CVP infections was significantly lower in patients with malignancies and those who received Orphis CV Kit Neo by both univariate and multivariate analyses (Table 3).

**Table 2:** Intraoperative and postoperative complications.

	Number	(%)
**Intraoperative complications**		
Arterial bleeding	7	2.2
Pneumothorax	4	1.3
**Postoperative complications**		
Infection	27	8.8
Extravasation	5	1.6
Obstruction	4	1.3
Deslocation	2	0.6
Catheter fracture	1	0.3
Evulsion of CVP	39	12.7

**Table 3:** Univariate and multivariate analyses to identify variables that are associated with CVP infection.

Variable	Factor	Univariate analysis			Multivariate analysis		
		Infection		p value	Odds ratio	95% CI	p value
		No	Yes				
Gender	Female	144 (90.6)	15 (9.4)	0.67	1	Reference	0.78
	Male	137 (92.0)	12 (8.0)		0.88	0.38–2.06	
Age		68.8 ± 11.2	71.1 ± 14.8	0.70			
Primary disease	Benign	25 (71.4)	10 (28.6)	0.0002	1	Reference	< 0.0001
	Malignant	256 (93.8)	17 (6.2)		0.13	0.05–0.34	
Puncture site	Internal jugular	200 (92.2)	17 (7.8)		1	Reference	
	Subclavian	77 (89.5)	9 (10.5)	0.33	0.66	0.25–1.77	0.41
	Others	4 (80.0)	1 (20.0)		0.28	0.02–3.39	0.32
Port device	Bard X-Port isp	86 (85.2)	15 (14.8)	0.008	1	Reference	
	Orphis CV Kit Neo	168 (95.5)	8 (4.5)		0.19	0.07–0.54	0.002
	Others	27 (87.1)	4 (12.9)		0.81	0.23–2.88	0.75

*CI* confidence interval.

In the present study, 39 patients (12.7%) experienced CVP evulsion due to postoperative complications (Table 2). Identification of risk factors associated with CVP evulsion is clinically important as the patients undergo unnecessary surgery to replace the CVP, which extends the treatment period. As shown in Table 4, both the type of primary disease and the type of device were associated with CVP evulsion in univariate and multivariate analyses. Malignancy (odds ratio 0.23, *p* = 0.002) and Orphis CV Kit Neo (odds ratio 0.18, *p* < 0.0001) were independently associated with a lower incidence of CVP evulsion.

**Table 4:** Univariate and multivariate analyses to identify variables that are associated with CVP evulsion.

Variable	Factor	Univariate analysis			Multivariate analysis		
		Evulsion of CVP		p value	Odds ratio	95% CI	p value
		No (n = 281)	Yes (n = 39)				
Gender	Female	136 (85.5)	23 (14.5)	0.32	1	Reference	0.37
	Male	133 (89.3)	16 (10.7)		0.72	0.35–1.47	
Age		68.9 ± 11.3	69.9 ± 12.9	0.61			
Primary disease	Benign	25 (71.4)	10 (28.6)	0.003	1	Reference	0.002
	Malignant	244 (89.4)	29 (10.6)		0.23	0.09–0.59	
Puncture site	Internal jugular	191 (88.0)	26 (12.0)		1	Reference	
	Subclavian	74 (86.1)	12 (13.9)	0.56	0.55	0.24–1.28	0.17
	Others	4 (80.0)	1 (20.0)		0.28	0.02–3.14	0.30
Port device	Bard X-Port isp	79 (78.2)	22 (21.8)	0.0003	1	Reference	
	Orphis CV Kit Neo	164 (93.2)	12 (6.8)		0.18	0.08–0.43	< 0.0001
	Others	26 (83.9)	5 (16.1)		0.65	0.21–1.98	0.45

*CI* confidence interval.

**Figure 1:**
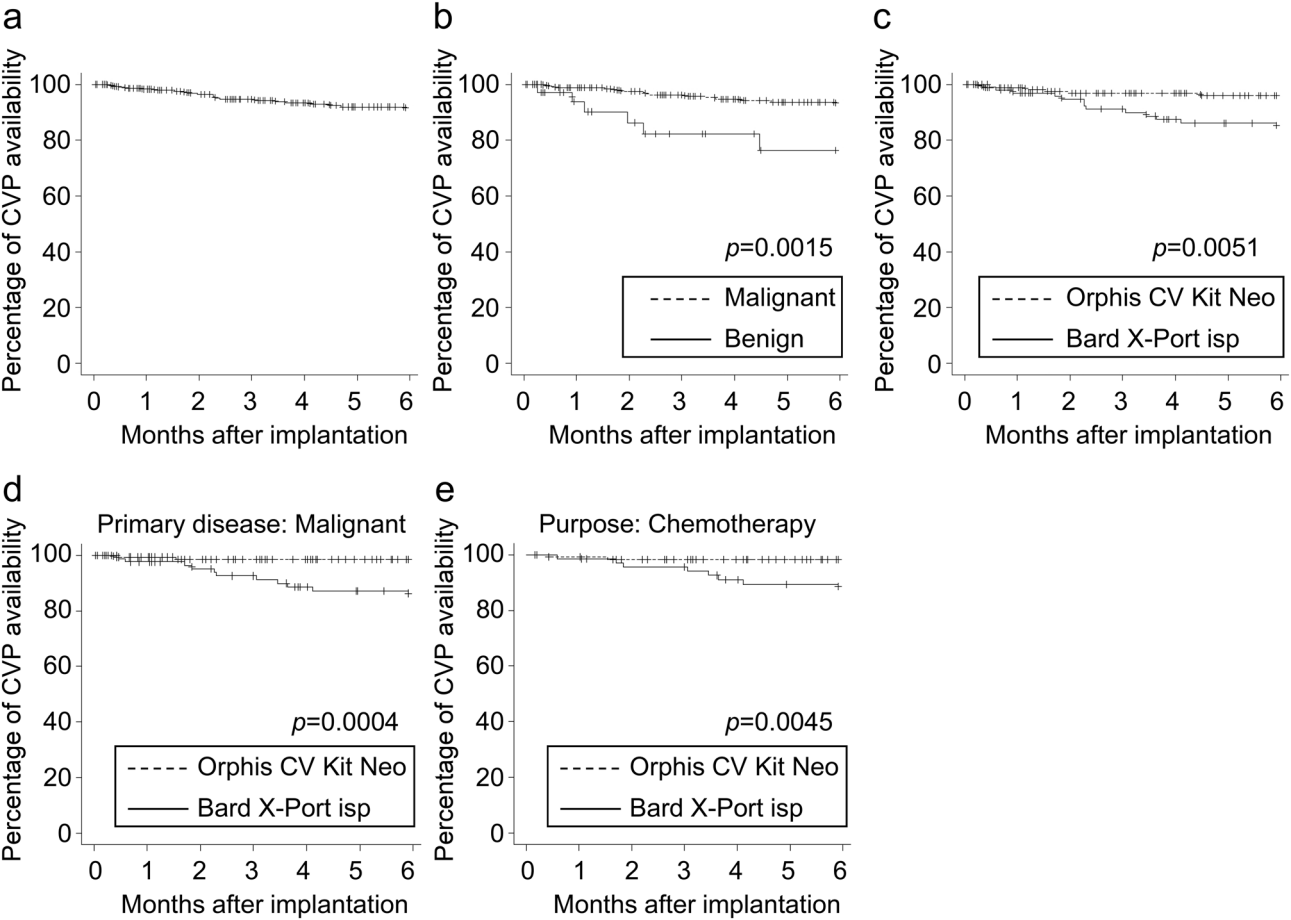
Kaplan–Meier curves showing central venous port (CVP) availability in (**a**) all patients, (**b**) patients with malignant or benign disease, and (**c**) patients with Orphis CV Kit Neo or Bard X-Port isp. Kaplan–Meier curves showing CVP availability in (**d**) patients with malignancies or (**e**) patients implanted for chemotherapy. Significance determined by the log-rank test is shown.

We also determined the long-term availability of CVP after implantation. The CVP availability at six months was 91.4% in the overall cohort (Fig. 1a). In agreement with the results related to the lower risk of CVP evulsion, the CVP availability at six months was higher in patients with malignancies compared to those with benign diseases (Fig. 1b, 93.1% vs 76.3%, *p* = 0.0015). Similarly, the CVP availability at six months was higher in patients who received Orphis CV Kit Neo compared to those who received Bard X-Port isp (Fig. 1c, 95.9% vs 84.6%, *p* = 0.0051). Importantly, in multivariate analysis, malignancy (hazard ratio 0.24, *p* = 0.006, Table 5) and the use of Orphis CV Kit Neo (hazard ratio 0.27, *p* = 0.015) were independent factors associated with longer CVP availability. We further investigated to identify patients that are more beneficial of using Orphis CV Kit Neo. The use of Orphis CV Kit Neo was associated with longer CVP availability in patients with malignancies (*p* = 0.0004, Fig. 1d) or those implanted for chemotherapy (*p* = 0.0045, Fig. 1e), but not in those with benign disease (*p* = 0.84) or those implanted for nutrition (*p* = 0.37), suggesting that Orphis CV Kit Neo would be more beneficial in patients with malignancies or those intended to use for chemotherapy.

**Table 5:** Univariate and multivariate analyses of long-term availability of CVP.

Variable	Factor	Number of patients	Availability at six months	Univariate analysis			Multivariate analysis		
				HR	95% CI	p value	HR	95% CI	p value
Gender	Female	159	91.7%	1	Reference	0.78	1	Reference	0.62
	Male	149	91.1%	1.13	0.48–2.66		1.25	0.52–2.96	
Age		308		1.01	0.97–1.05	0.85			
Primary disease	Benign	36	76.3%	1	Reference	0.003	1	Reference	0.006
	Malignant	272	93.1%	0.24	0.10–0.63		0.24	0.09–0.67	
Puncture site	Internal jugular	217	93.3%	1	Reference		1	Reference	
	Subclavian	86	87.3%	1.64	0.67–4.02	0.28	0.92	0.34–2.47	0.87
	Others	5	80.0%	6.00	0.76–47.1	0.09	1.23	0.13–12.0	0.86
Port device	Bard X-Port isp	101	84.6%	1	Reference		1	Reference	
	Orphis CV Kit Neo	176	95.9%	0.28	0.10–0.73	0.009	0.27	0.09–0.79	0.02
	Others	31	90.0%	0.53	0.12–2.34	0.40	0.49	0.11–2.26	0.36

*HR* hazard ratio, *CI* confidence interval.

## Discussion

Previous studies investigating risk factors for complications related to CVP implantation revealed that complications occurred in up to 33% of patients and that infections were the most common complication, ranging between 0.6% and 27%^1^. The reported potential risk factors for infection are benign disease (vs malignancy)^4^, hospitalized patients (vs outpatients)^7^, chemotherapy in an non-adjuvant setting (vs chemotherapy in an adjuvant setting)^8^, malignancy with metastasis (vs localized malignancy)^3^, approach from the femoral vein^7^, administration of total parenteral nutrition^5,6^, no antibiotic prophylaxis^5,9^, blood transfusion^3^, and chronic steroid use^5,10^. In accordance with these findings, the present study demonstrated that malignancy was significantly associated with lower rate of CVP infection and evulsion and longer CVP availability. Although the efficacy of antibiotic prophylaxis for CVP implantation was controversial around the start of the study period, intravenous antibiotic prophylaxis was initiated immediately before surgery in all patients who were relatively immunocompromized and thus were susceptible to infection following the implanting of an artificial foreign body. In fact, later studies have demonstrated a reduction in the CVP infection rate using antibiotic prophylaxis^5,9^.

We selected the internal jugular and subclavian veins as puncture sites in most of the study patients. Arterial puncture occurs in 6.3–9.4% of patients in whom CVP is planted in the internal jugular vein without ultrasound guidance; therefore, CVP placement with ultrasonic guidance is highly recommended^7,11^. Previous studies reported that the incidence rates of arterial puncture using the internal jugular and subclavian veins were 1.4–1.7% and 3.1–4.9%, respectively^11^. In the current study, arterial puncture occurred in 2.2% (7/308) of the patients, in agreement with previous studies. Pneumothorax or hemothorax are other major complications related to the puncture site. In the present study, 1.3% (4/308) of the patients developed pneumothorax, including 3.5% (3/86) of the patients undergoing the subclavian vein approach and 0.5% (1/217) of the patients undergoing the internal jugular vein approach. Notably, one patient undergoing the subclavian vein approach experienced tension pneumothorax. Previous studies reported that the incidence of pneumothorax ranged from 0.45 to 3.1% with the subclavian vein approach and was less than 0.2% with the internal jugular vein approach^11^. Accordingly, we have gradually shifted from the subclavian vein approach to the internal jugular vein approach. In some studies, CVP is implanted in the upper arm or forearm by approaching from the basilic or axillary veins to reduce puncture-associated complications^2^, and the reported CVP infection rates in patients undergoing the upper arm or forearm approach range from 2.9 to 9.9%. Approaching from the femoral vein increases the risk of infection and arterial puncture^7,11^; therefore, femoral vein is not usually selected as the initial site of approach. Selection of the puncture site differs among clinicians, since no approach is definitively superior to others^11,12^. Hence, it is important that the surgeons should select the approach in which they are most proficient.

In the present study, the use of Orphis CV Kit Neo was associated with a lower rate of infection and evulsion and with longer CVP availability compared with Bard X-Port isp. The device type was based on the physician’s discretion; however, there was a tendency to use Orphis CV Kit Neo in the latter years of the study. To eliminate the chronological effect on CVP evulsion, we compared the rate of CVP evulsion between the first and the second half of the cases. The percentage of CVP evulsion was not statistically different between patients with Bard X-Port isp (19.6% in the first half [n = 51] vs 24.0% in the second half [n = 50], *p* = 0.64) and those with Orphis CV Kit Neo (8.0% in the first half [n = 88] vs 5.7% in the second half [n = 88], *p* = 0.77), demonstrating the minimal involvement of this bias in the study results. The notable difference between the two devices is the biocompatible 2-methacryloyloxyethyl phosphorylcholine (MPC) polymer coating on the surface of Orphis CV Kit Neo, which was not present on the surface of Bard X-Port isp. The MPC coating mimics a biomembrane and reduces the adhesion of proteins, cells, and bacteria^13,14^. Several studies have demonstrated that MPC coating on dentures inhibits plaque deposition^13,14^. These studies, together with the current study findings, suggest that MPC coating on artificial implants including CVPs might reduce plaque deposition and infection.

The present study has several limitations. First, this was a retrospective study from a single center. Second, several potential risk factors such as blood transfusion and steroid use were not included in the analyses. Third, although the study results suggest the preventive potential of device type on CVP infection and evulsion, the underlying mechanisms involved, particularly the role of MPC coating, were not elucidated.

## Conclusion

The present retrospective study revealed that malignancy and the use of Orphis CV Kit Neo were significantly associated with a lower rate of infection and evulsion and with longer CVP availability.　The association of the use of Orphis CV Kit Neo with low infection rate suggests the preventive role of MPC coating in CVP infection.

## Methods

### Participants and methods

We retrospectively reviewed the medical records of 308 patients who underwent CVP placement at Yaizu City Hospital between January 2011 and December 2017. Patients who underwent replacement surgeries after CVP evulsion were excluded from the study. The clinical features of the patients, such as patient demographics, primary disease and treatment, surgical procedures, type of port device, CVP complications, and long-term CVP availability, were obtained from the medical records. The review board in Yaizu City Hospital approved the study.

The surgical procedure for CVP placement basically involved access to the internal jugular vein or subclavian vein under superficial ultrasonic guidance, and a port was placed in the precordial region. The approach was selected based on the patient’s condition and at the discretion of the primary physician. The devices used in this study were Bard X-Port isp (Medicon, Osaka, Japan, n = 101), Orphis CV Kit Neo (Sumitomo Bakelite, Tokyo, Japan, n = 176), and others (n = 31). Bard X-Port isp is a silicone rubber catheter without surface coating. Orphis CV Kit Neo has a biocompatible MPC polymer coating on the surface to prevent protein and cell adhesion; the catheter also contains an inner mesh to prevent catheter kinking and fracture. All placements were performed according to the manufacturers’ instructions under the supervision of board-certified surgeons. Antibiotic prophylaxis was intravenously administered immediately before the CVP placement in all patients. Informed consents were obtained from all patients to perform CVP placement. All methods were carried out in accordance to the principles set out in the World Medical Association Declaration of Helsinki and the National Institute of Health Belmont Report.

### Statistical analysis

Continuous variables were assessed using Student’s *t* test or Wilcoxon rank-sum test, and categorical variables were assessed using the χ^2^ test or Fisher’s exact test. Long-term CVP availability was analyzed using the Kaplan–Meier method with the log-rank test. Multivariate analysis was performed using the logistic regression model and the Cox proportional hazard model. All statistical analyses were performed with JMP version 11.0 (SAS Institute, Cary, NC) or R (The R Foundation for Statistical Computing, Vienna, Austria, version 3.6.1), and a two-sided *p* value of < 0.05 was considered to indicate statistical significance.

### Ethical approval

All methods were carried out in accordance to the principles set out in the World Medical Association Declaration of Helsinki and the National Institute of Health Belmont Report. Research involving Human Participants and/or Animals: Yes. Informed consent: Yes.
